# Heart Failure and Worsening Renal Function: Prevalence, Risk Factors, and Impact on Hospital Readmissions in an Urban Minority Population †

**DOI:** 10.3390/jcm14030877

**Published:** 2025-01-28

**Authors:** Asmaa AlShammari, Mariel Magdits, Rosemarie Majdalani, Sriraman Devarajan, Anna Hughes, Lily McCann, Natalia Ionescu, Farbod Raiszadeh

**Affiliations:** 1 Kuwait’s Foundation for the Advancement of Sciences, Kuwait City 12081, Kuwait; 2 The Institute of Human Nutrition, Columbia University Irving Medical Center, New York, NY 10032, USA; 3 Harlem Hospital Center, Health + Hospitals, Vagelos College of Physicians and Surgeons, Columbia University, New York, NY 10037, USAraiszadf@nychhc.org (F.R.); 4 Dasman Diabetes Institute, Kuwait City 15462, Kuwait

**Keywords:** heart failure, minority groups, risk factors, patient readmission, epidemiology

## Abstract

**Background and Objectives**: Heart failure (HF) often leads to worsening renal function (WRF), negatively impacting patient outcomes. This study aims to examine the incidence of WRF in HF patients, identify its risk factors, and assess its effect on readmissions. **Materials and Methods**: This retrospective analysis included 297 HF patients admitted to Harlem Hospital Center between January 2019 and December 2021. WRF incidence and its association with risk factors, hospital stays, and readmissions were analyzed. Data on age, type 2 diabetes, chronic kidney disease, high-dose furosemide use, and biomarkers (ProBNP, troponin T, creatinine) were collected. A risk-scoring system was developed to identify patients at higher risk for WRF. **Results**: WRF occurred in 27% of patients, with a significant correlation to longer hospital stays and lower cardiology follow-up adherence. Risk factors for WRF included older age, type 2 diabetes, chronic kidney disease, high-dose furosemide, and elevated ProBNP, troponin T, and creatinine levels. The risk scoring system revealed that patients scoring 6 or higher were four times more likely to develop WRF. Interestingly, WRF did not increase 30-day readmission rates. **Conclusions**: This study highlights the high incidence of WRF among HF patients, its impact on hospital stays and follow-up adherence, and the utility of a risk-scoring system to identify vulnerable patients. The findings offer valuable insights into improving care in minority-serving hospitals and provide a foundation for future research on WRF in HF patients.

## 1. Introduction

Heart failure (HF) affects approximately 6.5 million adults in the United States, with projections indicating a 46% increase by 2030. It remains a leading cause of hospitalization among older adults [1,2]. Worsening renal function (WRF), defined as a 0.3 mg/dL increase in serum creatinine from baseline, is commonly observed in HF patients and has been associated with adverse outcomes, including longer hospital stays and increased cardiovascular mortality [3,4]. Although WRF is a known predictor of poor prognosis in HF patients, studies focusing on WRF predictors, particularly in Black/African American populations, are scarce [2,5,6].

Black/African American individuals experience a higher prevalence of HF and tend to present with symptoms earlier than other populations. Previous reports indicate that Black patients with HF and chronic kidney disease (CKD) have a higher progression rate of CKD, which increases their risk of End-Stage Kidney Disease (ESKD) [7,8,9]. However, the underlying factors that drive this accelerated progression remain unclear. Harlem Hospital Center (HHC) serves a predominantly African American and Hispanic patient population, many of whom face socioeconomic challenges. Heart disease, including HF, is a major contributor to mortality and health disparities in this population [10]. Despite the high burden of HF and its association with WRF in this group, little is known about the specific risk factors that contribute to WRF in these patients. Understanding WRF in this context is crucial for improving patient outcomes and addressing healthcare disparities. Specifically, the role of socioeconomic factors, comorbid conditions, and access to care in predicting WRF among Black/African American and Hispanic populations at HHC is still not well defined.

This study aims to determine the prevalence and predictors of WRF among Congestive Heart Failure (CHF) patients at HHC, offering actionable insights for risk identification and management. It also evaluates short-term readmission rates linked to WRF, contributing to improved detection and prevention strategies. Building on Forman et al. [11], we explore additional risk factors and assess the applicability of previous findings to our predominantly Black/African American and Hispanic population at HHC. The goal is to develop tailored prevention strategies and address healthcare disparities in this vulnerable community, where the unique factors contributing to WRF remain underexplored.

## 2. Materials and Methods

This retrospective study was approved by the Harlem Hospital Institutional Review Board (IRB) as part of the CHF Registry Baseline Data project, which is aimed at future performance improvement initiatives. Data were collected from January 2019 to December 2020, focusing on all known CHF patients admitted to Harlem Hospital Center. Inclusion Criteria included age of 18 years or older with a primary diagnosis of CHF or with a CHF-related hospitalization. Exclusion criteria included severe cardiac disorders (e.g., valvular disease, severe aortic stenosis), end-stage kidney disease requiring chronic dialysis (as further worsening of renal function would not occur), and hospital stays of less than two days.

Although CHF diagnosis was identified through multiple databases at Harlem Hospital Center, including the electronic medical record system (EPIC), a code list managed by the Care Management Department, and the monthly billing list from Get with the Guidelines (GWTG-HF), diagnosis confirmation was achieved through a comprehensive review of cardiology consultations, medical team notes, laboratory test results, and echocardiograms. After screening for exclusions, 297 patients were included in the final analysis: 216 without WRF and 81 with WRF. 

Key variables included demographic characteristics, comorbidities (e.g., diabetes, CKD), laboratory values (e.g., creatinine, ProBNP, troponin T), medications (e.g., furosemide), and hospital readmission data. WRF was defined as a ≥ 0.3 mg/dL increase in serum creatinine from admission. Data completeness was assessed for several key variables: income source data were available for 293 out of 297 patients (98.7% complete, 1.3% missing), left ventricular ejection fraction (LVEF) data were available for 280 out of 297 patients (94.3% complete, 5.7% missing), and body mass index (BMI) data were available for 293 out of 297 patients (98.7% complete, 1.3% missing). Missing data for these variables were handled as part of the statistical analysis.

The primary outcomes of this study included the prevalence of WRF in CHF patients, the identification of risk factors associated with WRF, the impact of WRF on hospital length of stay, and the effect of WRF on 30-day readmission rates. Secondary outcomes encompassed adherence to follow-up appointments and differences in medication use, particularly diuretics, between WRF and No WRF groups.

Comparative analyses between the WRF and the No WRF group were performed using Fisher’s Exact Test for categorical variables, the Wilcoxon Rank-Sum Test for continuous or ordinal variables with non-normal distributions, and the chi-square test for categorical variables with adequate expected frequencies. These methods were chosen to ensure robust and appropriate handling of the dataset’s characteristics. Multicollinearity among independent variables was assessed using variance inflation factors (VIF), with a VIF > 10 indicating high multicollinearity.

Confounding variables for the multivariate analysis were selected systematically. Univariate analyses were utilized to identify variables significantly associated with WRF. Multivariable Cox regression models were used to identify independent predictors of WRF, with a risk score constructed based on multivariate-adjusted risk ratios. To assess model stability, a bootstrap sampling method was applied. Given the non-parametric nature of the data, this approach was deemed more reliable for inference.

All statistical analyses were two-sided and conducted using SPSS version 25 (SPSS Inc., Chicago, IL, USA). Building on the methodological approach of Forman et al. (2004), this study aimed to explore risk factors for WRF in a specific patient population [11]. While Forman et al. identified key predictors of worsening renal function in heart failure patients, this study seeks to understand the risk factors relevant to our predominantly Black/African American and Hispanic patient population at Harlem Hospital Center.

## 3. Results

### 3.1. Demographics and Clinical Data

Table 1 presents the characteristics of the 297 CHF patients (mean age: 64; 57.2% male). A large majority of patients were from low-income backgrounds, with 81.8% being Medicare/Medicaid beneficiaries. Black/African American patients comprised 75.4% of the cohort. WRF patients were significantly older and had higher Left Ventricular Ejection Fractions (LVEF), although no difference in LVEF severity was observed between groups. Abnormal troponin levels were more common in WRF patients (69.1% vs. 48.1%), and a higher percentage had admission serum creatinine ≥ 1.5 mg/dL.

### 3.2. Hospital Length of Stay and Furosemide Use

Patients with WRF had a longer hospital length of stay and were prescribed higher doses of intravenous and oral furosemide compared to those without WRF. Table 2 illustrates these differences, highlighting the increased use of diuretics among the WRF group.

### 3.3. Biomarkers of WRF

In Table 3, hemoglobin A1C%, phosphorus, and creatinine levels were significantly higher in patients with WRF, while ProBNP and hemoglobin levels were higher in the No WRF group. No significant differences in potassium levels were observed between the two cohorts.

### 3.4. Follow-Up and Readmission

Follow-up data showed that patients with WRF were less likely to attend follow-up appointments, as shown in Table 4. However, there was no significant difference in 30-day readmission rates between WRF and No WRF patients.

### 3.5. Key Risk Factors for WRF in CHF Patients

Table 5 shows minimal bias with key risk factors for worsening renal function (WRF) in CHF patients, including age ≥65 years, type 2 diabetes, CKD, elevated Troponin T (≥0.04 ng/mm), elevated ProBNP (≥9000 ng/mL), admission creatinine levels (1.5–2.5 mg/dL and >2.5 mg/dL), and daily furosemide dosage (>100 mg/day, oral and IV). For ProBNP, an arbitrary cutoff was selected based on the dataset’s mean values, while Troponin and Creatinine cutoffs were based on standard values established in the existing literature.

### 3.6. Risk Score Development

Table 6 presents a risk score incorporating key risk factors for WRF: oral furosemide use (1 point), creatinine >2.5 mg/dL (3 points), and other factors (2 points). Patients with a risk score ≥ 6 were found to have a fourfold increased likelihood of developing WRF compared to those with a score of 0. This risk stratification tool, based on readily available clinical variables, has potential utility in identifying high-risk patients and guiding preventive interventions. Future studies should validate the score in independent cohorts and evaluate its implementation in clinical workflows.

## 4. Discussion

This study contributes to ongoing efforts to evaluate the inpatient and post-discharge experiences of patients with congestive heart failure (CHF) at Harlem Hospital Center, with a focus on addressing health disparities in underserved areas such as Harlem. The high prevalence of chronic diseases like diabetes, cardiovascular disease, and obesity among minority communities is closely linked to rehospitalization rates and poor prognosis, emphasizing the need for targeted interventions [10].

These findings both confirm and extend the work of Forman et al. (2004), identifying similar risk factors for worsening renal function while providing new insights specific to our predominantly Black/African American and Hispanic populations [11]. The variations in our risk profile underscore the importance of population-specific research and highlight the need for tailored prevention strategies in minority-serving healthcare settings.

The study found that 27.3% of CHF patients at Harlem Hospital Center developed worsening renal function (WRF). The multifactorial nature of WRF in CHF is well-documented, including hemodynamic imbalances, neurohormonal activation, medication effects, and inflammatory processes [6,7,12]. Key baseline characteristics associated with WRF were identified, including demographic (age), systemic/hemodynamic (diabetes, elevated BUN), renal (high baseline creatinine, chronic kidney disease (CKD)), medication-related (loop diuretics), and cardiac (high ProBNP, Troponin T) factors (Table 5). We developed a predictive point score based on these factors, showing that patients with a score of 6 or higher were four times more likely to develop WRF compared to those with a score of 0.

These findings align with previous studies on heart failure populations, confirming the clinical relevance of T2DM and CKD as independent risk factors for WRF through Cox regression analysis (Table 1). Moreover, the strong link between CKD and cardiovascular death, especially in Black/African American populations, highlights the need for tailored interventions targeting these comorbidities in CHF management [7,13,14].

Medications, particularly loop diuretics, also played a role in WRF progression. While diuretics improve heart failure symptoms, they can reduce renal function by activating the Renin-Angiotensin-Aldosterone-System (RAAS) [13,15]. Our findings suggest that higher doses of furosemide were associated with WRF, raising questions about optimal dosing strategies and the need for individualized diuretic management [16].

Elevated biomarkers like ProBNP (≥9000 ng/dL) and Troponin T (≥0.04 ng/mm) were found to be prognostic indicators of WRF, with a potential bi-directional relationship implicated by venous congestion [17,18,19,20,21,22]. Furthermore, elevated creatinine levels on admission (1.5–2.5 mg/dL) were identified as independent risk factors for WRF (*p* < 0.001), which corresponds with the longer hospital stays, higher costs, and increased mortality rates associated with renal dysfunction in CHF [4]. While baseline potassium levels showed no differences in our study, the literature links hyperkalemia to faster renal decline, worse outcomes in heart failure, and higher risks with comorbidities like diabetes and stroke [23,24,25]

Interestingly, no significant difference was observed in 30-day readmissions between WRF and No-WRF groups (Table 5), suggesting that WRF alone may not be a decisive factor in short-term outcomes [26]. One possible explanation for the lack of an observed association between WRF and 30-day readmissions is that factors unique to our population, such as differences in care delivery, discharge planning, and follow-up practices, may mitigate this impact. Our data reveal a significant gap in timely follow-up appointments, which could lead to uniformly high readmission rates regardless of renal function status. Additionally, since our study only includes readmissions to Harlem Hospital Center, it does not capture those to other healthcare facilities, potentially underestimating the true readmission rate. Further, the high prevalence of comorbidities, such as diabetes and CKD, may predispose patients to frequent rehospitalizations, independent of WRF, further diluting the observed relationship.

Beyond clinical markers, our study also highlighted healthcare utilization patterns, such as prolonged hospital stays associated with WRF, and the need for improved care coordination post-discharge. Notably, a significant proportion of patients lacked timely follow-up appointments, underscoring the need for enhanced discharge planning and post-discharge monitoring to reduce readmission rates [27,28].

Our findings in this predominantly-minority population both align with and diverge from broader heart failure cohorts. While we observed similar risk factors for WRF, such as diabetes, chronic kidney disease, and elevated biomarkers, we noted a higher WRF prevalence (27.3%) compared to the 20–25% typically reported. This difference may reflect the higher comorbidity burden in our predominantly Black/African American and Hispanic population. Recent evidence indicates racial variations in heart failure drug efficacy, with SGLT2 inhibitors offering greater benefits for black patients with HFrEF, while beta-blockers may have less impact on mortality in this group. These findings underscore the importance of considering racial and ethnic differences in drug response when tailoring heart failure therapies [29]. 

### 4.1. Clinical Implications

The identification of key risk factors for WRF—such as diabetes, CKD, and elevated biomarkers—can inform clinical practice by enabling earlier detection and personalized management strategies for high-risk CHF patients. Additionally, optimizing medication regimens and improving care coordination at discharge may help reduce the risk of WRF and readmissions, ultimately enhancing patient outcomes and reducing healthcare costs.

### 4.2. Strengths and Limitations

Our study’s strengths include the focus on a high-risk, predominantly minority population and the development of a predictive point score for WRF, which can inform clinical decision-making. However, limitations include its retrospective design and focus on a single institution, which limits generalizability. Furthermore, the absence of long-term follow-up data, including mortality and extended readmissions, warrants future investigation. In addition, we acknowledge the lack of data on domiciliary treatment as a limitation, as it prevents us from evaluating its potential impact on patient outcomes.

### 4.3. Future Directions

Future research should explore the long-term outcomes of WRF in CHF patients, including mortality and quality of life. Studies evaluating interventions to prevent or mitigate WRF, particularly those targeting high-risk subgroups, are also needed. Additionally, examining strategies to improve post-discharge care, including follow-up appointments and medication management, could reduce readmissions and enhance patient outcomes.

## 5. Conclusions

This study provides valuable insights into the prevalence, risk factors, and healthcare utilization patterns associated with WRF in CHF patients. By identifying critical factors such as diabetes, CKD, and elevated biomarkers, we emphasize the importance of tailored interventions to manage comorbidities and improve treatment outcomes. While the study sheds light on healthcare utilization challenges, further research is needed to evaluate long-term outcomes and explore interventions that can optimize patient care and reduce disparities in minority communities.

## Figures and Tables

**Table 1:** Demographic and clinical characteristics of patients in the CHF registry.

Characteristics	All Patients n = 297	WRF n = 81 (27%)	No WRF n = 216 (73%)		*p* Value
Age, years, mean ± SD	64 ± 13.0	67 ± 14.0	63 ± 12.0		0.008
LV ejection fraction %, mean ± SD	38 ± 14.0	41.1 ± 15.0	36 ± 12.9		0.043
Males, n (%)	170 (57.2%)	50 (61.7%)	120 (55.6%)		0.359
BMI, Kg/m^2^					
Underweight (<18.5)	6 (2.0%)	1 (1.2%)	5 (2.3%)		1.000
Normal Weight (18.5–24.9)	72 (24.2%)	23 (28.4%)	49 (22.7%)		0.362
Overweight (25–29.9)	73 (24.6%)	28 (34.6%)	45 (20.8%)		0.023
Obesity Class I (30–34.9)	52 (17.5%)	8 (9.9%)	44 (20.4%)		0.039
Obesity Class II (35–39.9)	33 (11.1%)	9 (11.1%)	24 (11.1%)		1.000
Obesity Class III (>40)	53 (17.8%)	12 (14.8%)	41 (19.0%)		0.497
Missing	8 (2.7%)	0 (0.0%)	8 (3.7%)		0.113
Insurance Plan, n (%)					
Medicare	112 (37.7%)	32 (39.5%)	80 (37.0%)		0.689
Medicaid	131 (44.1%)	35 (43.2%)	96 (44.4%)		0.896
Other	34 (11.4%)	8 (9.9%)	26 (12.0%)		0.686
Uninsured	6 (2.0%)	1 (1.2%)	5 (2.3%)		1.000
More than one source	14 (4.7%)	5 (6.2%)	9 (4.2%)		0.539
Race/Ethnicity					
White	7 (2.4%)	3	4	1.9	0.395
Black or African American	222 (74.7%)	61	161	74.5	1.000
Hispanic or Latino	38 (12.8%)	11	27	12.5	0.846
Others/Unknown	30 (10.1%)	6	24	11.1	0.396
Income					
Employer	31 (10.4%)	7 (8.6%)	24 (11.1%)		0.671
Social Security	104 (35.0%)	32 (39.5%)	72 (33.3%)		0.341
Government Assistance	5 (1.7%)	2 (2.5%)	3 (1.4%)		0.616
Family Support	21 (7.1%)	7 (8.6%)	14 (6.5%)		0.611
More than one source *	43 (14.5%)	7 (8.6%)	36 (16.7%)		0.096
None/Unable to Determine	90 (30.3%)	23 (28.4%)	67 (31.0%)		0.777
Missing	3 (1.0%)	3 (3.7%)	0 (0.0%)		0.020
Smoking					
Current Smoker	98 (33.0%)	26 (32.1%)	72 (33.3%)		0.890
No/Unable to Determine	84 (28.3%)	24 (29.6%)	60 (27.8%)		0.773
Missing	115 (38.7%)	31 (38.3%)	84 (38.9%)		1.000
Alcohol Use					
Current Alcohol use	67 (22.6%)	8 (9.9%)	59 (27.3%)		0.001
No/Unable to Determine	34 (11.4%)	10 (12.3%)	24 (11.1%)		0.838
Missing	196 (66.0%)	63 (77.8%)	133 (61.6%)		0.009
LV Ejection Fraction, %					
Normal (M: >52%, F: >54%)	26 (8.8%)	10 (12.3%)	16 (7.4%)		0.247
Mild (M: 41–51%, F: 41–53%)	49 (16.5%)	18 (22.2%)	31 (14.4%)		0.115
Moderate (30–40%)	88 (29.6%)	22 (27.2%)	66 (30.6%)		0.669
Severe (<30%)	5 (1.7%)	0 (0.0%)	5 (2.3%)		0.328
Missing	129(43.4%)	31 (38.3%)	98 (45.4%)		0.295
Serum Creatinine, mg/dL					
<1.5	196 (66.0%)	42 (51.9%)	154 (71.3%)		0.002 0.067
1.5–2.5	56 (18.9%)	21 (25.9%)	35 (16.2%)		
≥2.5	45 (15.1%)	18 (22.0%)	27 (12.5%)		
Comorbidities, n (%)					
CKD	163 (54.9%)	52 (64.2%)	111 (51.4%)		<0.001
Obesity	138 (46.5%)	29 (35.8%)	109 (50.5%)		0.023
Hypertension	269 (90.6%)	75 (92.6%)	194 (89.8%)		0.656
Type 1 Diabetes	15 (5.1)	3 (3.7%)	12 (5.6%)		0.578
Type 2 Diabetes	177 (59.6%)	61 (75.3%)	116 (53.7%)		0.001
Dyslipidemia	136 (45.8%)	36 (44.4%)	100 (46.3%)		0.795
Coronary artery disease	92 (31.0%)	24 (29.6%)	68 (31.5%		0.780

Values are given as mean ± SD for continuous variables and as number (percentage) for categorical variables. * Patients can have multiple sources of income. WRF, worsening renal function; LV, left ventricle; BMI, body mass index; CKD, chronic kidney disease defined as non-dialysis dependent chronic kidney disease with estimated glomerular filtration rate (eGFR) ≤ 60 mL/min.

**Table 2:** In-hospital diuretic treatment.

Characteristics	All Patients n = 297	WRF n = 81 (27%)	No WRF n = 216 (73%)	*p* Value
Length of stay, days, mean ± SD, (range)	6 ± 2 (2–52)	9 ± 3 (2–39)	6 ± 2 (2–52)	0.001
IV Furosemide during hospital stay, mg/day, mean ± SD, (range)	141 ± 40 (20–720)	196 ± 52 (20–720)	125 ± 51 (20–560)	0.008
Oral Furosemide, mg/day, mean ± SD, (range)	154 ± 83 (20–800)	400 ± 92 (20–760)	160 ± 86 (20–800)	0.002
Oral Torsemide, mg/day, mean ± SD, (range)	39 ± 18.7 (10–90)	76 ± 12 (20–60)	41 ± 11 (10–90)	0.268
Oral Bumetanide, mg/day, mean ± SD, (range)	3.8 ± 2.7 (1–9)	4 ± 3 (1–5)	5 ± 3 (1–9)	0.368
Daily Creatinine change, mg/dl, mean ± SD, (range)	1.9 ± 1.0 (0.4–11)	2.8 ± 1.8 (0.7–11)	1.5 ± 0.9 (0.4–7)	<0.001

Values are given as mean ± SD for continuous variables and as number (percentage) for categorical variables. IV, intravenous.

**Table 3:** Laboratory Variables Collected During Hospitalization.

	WRF [n = 81]	No WRF [n = 216]	*p* Value
Lab Variables, mean ± SD			
WBC (×10^3^/mcL)	8.0 ± 3.5	8.2 ± 4.2	0.657
Hemoglobin (g/dL)	11.9 ± 2.4	11.0 ± 2.7	0.006
Glucose (mg/dL)	153.3 ± 76.5	176.9 ± 87.7	0.035
BUN (mg/dL)	24.3 ± 14.5	30.6 ± 17.5	0.006
Creatinine (mg/dL)	1.4 ± 0.9	2.0 ± 1.7	0.006
Phosphorus (mg/dL)	3.7 ± 1.0	3.9 ± 1.2	0.275
eGFR, Non-African American (mL/min/1.73 m^2^)	60.4 ± 33.2	49.3 ± 33.5	0.013
eGFR, African American (mL/min/1.73 m^2^)	72.7 ± 39.8	59.6 ± 40.4	0.015
proBNP (pg/mL)	7419.4 ± 10,464.7	12,149.4 ± 13,554.1	0.007
Hemoglobin A1C (%)	6.8 ± 1.7	7.4 ± 1.7	0.005
Troponin T (ng/mL)	0.0 ± 0.1	0.1 ± 0.4	0.184
Potassium (mmol/L)	4.5 ± 0.9	4.4 ± 0.7	0.442

Values are given as mean ± SD for continuous variables. WBC, white blood cells; Hgb, hemoglobin; BUN, blood urea nitrogen; eGFR, estimated glomerular filtration rate; BNP brain natriuretic peptide.

**Table 4:** Admissions, Readmissions, and Appointments of Patients in the CHF Registry.

	All Patients n = 297	WRF n = 81 (27%)	No WRF n = 216 (73%)	*p* Value
Follow-up appointment scheduled at HHC within 7 days of discharge, n (%)				
Yes within 7 days	72 (24.2%)	14 (17.3%)	58 (26.9%)	0.096
Longer than 7 days	134 (45.1%)	32 (39.5%	102 (47.2%)	0.242
Not scheduled	91 (30.6%)	35 (43.2%)	56 (25.9%)	0.005
Patient attended follow-up Harlem cardiology clinic appointment, n (%)				
Yes	90 (30.3%)	15 (18.5%)	75 (34.7)	0.007
No/Unable to determine	95 (32.0%)	35 (43.2%	60 (27.8%)	0.017
1st appointment future date	8 (2.7%)	1 (1.2%)	7 (3.2%)	0.688
No show	104 (35.0%)	30 (37.0%)	74 (34.3%)	0.683
Were any Harlem Cardiology Clinic appointments attended? n (%)				
Yes	127 (42.8%)	23 (28.4%)	104 (48.1%)	0.002
No	170 (57.2%)	58 (71.6%)	112 (51.9%)	
How many Harlem Cardiology Clinic appointments were attended? n (%)				
<5	102 (34.3%)	17 (21.0%)	85 (39.4%)	0.004
>5	25 (8.4%)	6 (7.4%)	19 (8.8%)	0.817
Missing	170 (57.2%)	58 (71.6%)	112 (51.9%)	0.002
Was the patient readmitted within 30 days of index hospitalization? n (%)				
Yes	44 (14.8%)	13 (16.0%)	31 (14.4%)	0.716
No	249 (83.8%)	65 (80.2%)	184 (85.2%)	0.295
Missing	4 (1.3%)	3 (3.7%)	1 (0.5%)	0.063
Readmission related to CHF, n (%)				
Yes	33 (11.1%)	11 (13.6%)	22 (10.2%)	0.412
No	11 (3.7%)	2 (2.5%)	9 (4.2%)	0.733
Missing	253 (85.2%)	68 (84.0%)	185 (85.6%)	0.716

Statistics presented: mean, SD; n, %

**Table 5:** Risk Factors of WRF.

Description	Parameter Estimate	Hazard Ratio	95% Confidence Interval ^1^		*p* value	Weight	Bootstrap Results (1000 Replicas)		
			Lower	Upper			Bias ^2^	Lower	Upper
Age ≥ 65	0.510	1.710	1.213	2.215	0.001	2	−0.005	0.228	0.665
T2DM	0.426	1.597	1.197	2.591	0.006	2	0.333	−0.033	1.001
CKD	0.453	1.813	1.203	2.737	0.002	2	−0.003	0.152	0.617
Troponin ≥ 0.04 ng/mL	0.480	1.654	1.254	2.171	<0.001	2	−0.003	0.332	0.713
ProBNP ≥ 9000 pg/mL	0.671	1.888	1.367	2.790	0.001	2	−0.003	0.369	0.881
1.5 ≤ creatinine mg/dL < 2.5	0.682	2.002	1.133	3.742	0.009	2	−0.022	−0.608	0.128
Creatinine ≥ 2.5 mg/dL	0.843	2.591	1.640	3.432	0.006	3	−0.044	−0.216	0.678
Furo_IV ≥ 100 mg/day	0.650	1.805	1.119	3.106	0.002	2	0.004	0.273	0.653
Furo_PO ≥ 100 mg/day	0.502	1.309	1.093	2.122	0.002	1	0.006	0.179	0.551

Cox regression with stepwise method using baseline as candidate variables ^1^ CI = Confidence Interval of hazard ratio ^2^ Bias from the original parameter estimate and 95% CI of the original parameter estimate. T2DM; type 2 diabetes mellitus; CKD, chronic kidney disease; BNP, brain natriuretic peptide; Furo, furosemide; IV, intravenous; PO, oral.

**Table 6:** WRF Risk Score.

Score, n (%)	Total Cohort 297	WRF Patients 81 (27%)	Relative Risk
N = 0	11 (3.7%)	1 (9.1%)	1 (Reference)
N = 1	19 (6.4%)	4 (21.1%)	1.32
N = 2	29 (9.8%)	7 (24.1%)	1.51
N = 3	35 (11.8%)	8 (22.9%)	2.26
N = 4	47 (15.8%)	10 (21.3%)	2.56
N = 5	54 (18.2%)	14 (25.9%)	3.02
N = 6+	102 (34.3%)	43 (42.2%)	3.78
*p*-value < 0.001			

## Data Availability

Data cannot be shared due to IRB stipulations and concerns regarding the privacy and confidentiality of study subjects.
